# BRASH Syndrome in the Absence of Chronic Kidney Disease: A Case Report

**DOI:** 10.5811/cpcem.38090

**Published:** 2025-02-15

**Authors:** Anthony Zaffino, Amanda Polsinelli, Adam Purdy

**Affiliations:** * Riverside Regional Medical Center, Emergency Department, Newport News, Virginia; † Riverside Regional Medical Center, Critical Care Department, Newport News, Virginia

**Keywords:** Case report, BRASH, shock

## Abstract

**Introduction:**

Bradycardia, Renal failure, Atrioventricular nodal blockade, Shock, Hyperkalemia (BRASH syndrome) is commonly misdiagnosed in the emergency department, which can lead to a delay in care and poor patient outcomes.

**Case Report:**

We present a case of BRASH syndrome in a patient with no underlying renal disease, which further complicated diagnosis and delayed treatment.

**Conclusion:**

Prompt recognition of the underlying pathophysiology in cases of BRASH syndrome is essential to guide treatment and avoid delays in care.

## INTRODUCTION

Bradycardia, Renal failure, Atrioventricular nodal blockade, Shock, Hyperkalemia (BRASH syndrome), first described in 2016, refers to a cascade of physiological abnormalities that is often misdiagnosed in the Emergency Department as either hyperkalemia or bradycardia. Anchoring solely on treating one of these aspects of the full syndrome is common and can lead to a delay in adequate care and worsening of the full clinical picture, with increasing likelihood of cardiovascular collapse in these patients.^1^ Understanding of the synergistic effects of hyperkalemia, atrioventricular nodal blockade, and renal failure allows timely and effective treatment of this syndrome. The following case describes a patient without chronic kidney disease who developed BRASH syndrome and severe hemodynamic instability. This case illustrates the importance of prompt recognition and holistic treatment of this novel syndrome, even in patients without underlying renal dysfunction.

## CASE REPORT

An 81-year-old female with a past medical history of hypertension, congestive heart failure, atrial fibrillation, diabetes mellitus, and asthma on twice daily 50 mg metoprolol and once daily 25 mg lisinopril presented to the emergency department via medics for evaluation of fatigue. The patient had a left total hip arthroplasty performed four days prior to arrival. She complained of fatigue over the two days prior to presentation. Upon arrival to the emergency department, the patient was notably fatigued to the point of requiring multiple verbal prompts to answer questions. She was oriented to person, place, and time. The patient denied any associated symptoms, solely complaining of severe fatigue. Initial vital signs included mild bradycardia at 57 beats per minute as well as hypotension with a blood pressure of 71/56 millimeters of mercury (mm Hg). On physical exam, mild pitting edema of the bilateral lower extremities was noted as well as mild wheezing in all lung fields. The patient was noted to be hypoxic with oxygen saturation of 88%. She was noted to have increased work of breathing and was placed on supplemental oxygen via nasal cannula with improvement to 95% oxygen saturation.

Patient’s workup revealed an acute kidney injury with a creatinine of 3.1 milligrams per deciliter (mg/dL) (reference range 0.44 – 1.03 mg/dL) from a baseline of 1.08 mg/dL per chart review (notably the reference range from that facility was 0.6–1.3 mg/dL). Pertinent lab values included a potassium of 5.5 millimoles per liter (mmol/L) (3.5 – 5.5 mmol/L), high sensitivity troponin of 393 picograms (pg)/mL (0 – 12 pg/mL), lactic acid of 3.1 mmol/L (0.5 – 2.0 mmol/L), beta natriuretic peptide of 736 pg/mL (0 – 100 pg/mL). Twelve lead electrocardiogram showed atrial fibrillation with slowed ventricular response. (Image) Her blood pressure initially improved to 90 mm Hg systolic after one liter normal saline bolus however her bradycardia worsened with the heart rate intermittently lowering into the 30s. One mg intravenous atropine was administered with no effect. While awaiting lab results, the patient’s mentation and blood pressure began to decline, leading to central line placement and administration of norepinephrine with subsequent improvement in both heart rate and blood pressure, though the patient remained mildly altered. Cardiology was consulted and with concern for unintentional overdose of metoprolol recommended holding this medication and continuing supportive care. The patient was then admitted to the intensive care unit for further management.

Overnight the patient’s renal function continued to decline despite intravenous fluid resuscitation. With concern for possible sepsis, blood cultures, respiratory culture, and urine culture were all collected, but all showed no growth at 48 hours. Her hospital course was complicated by a course of hyperkalemia (serum potassium level was 6.4 mmol/L) and she received standard treatments for hyperkalemia of calcium, insulin, dextrose, and sodium zirconium cyclosilicate. At this point, with low suspicion of septic shock and rising concern for BRASH syndrome, she received further intravenous fluids and was continued on vasopressor support. After five days of continued care focused on holding atrioventricular nodal blocking agents and medical treatment of hyperkalemia, the patient’s renal function returned to baseline and she was ultimately discharged home.

**CPC-EM Capsule**

What do we already know about this clinical entity?

*Bradycardia, renal failure, atrioventricular nodal blockade, shock, and hyperkalemia (BRASH) syndrome syndrome is a vicious cycle of worsening renal function and bradycardia, fueled in part by atrioventricular nodal blockade and hyperkalemia, eventually leading to cardiogenic shock.*

What is the major learning point?

*The image shows the severity of bradycardia that can be induced, while the figure shows a simplified flowchart of the pathophysiology of BRASH syndrome*

How might this improve emergency medicine practice?

*More awareness of this syndrome, in particular even in patients without underlying renal*

## DISCUSSION

Bradycardia, renal failure, atrioventricular nodal blockade, shock, and hyperkalemia syndrome is an emerging cascade of symptoms that can lead to significant morbidity and mortality if not correctly diagnosed and treated. The mixture of atrioventricular nodal blockade, often due to a beta blocker or calcium channel blocker, with renal disease leads to a vicious cycle of decreased clearance of said atrioventricular nodal blocker, which therefore leads to worsening bradycardia and ultimately cardiogenic shock. As cardiac output decreases, renal perfusion is further compromised, leading to a deterioration in renal function (Figure). Misdiagnosis of the etiology for the patient’s cardiogenic shock can lead to less optimal treatment and can literally be a fatal mistake. While it can be challenging to parse through the multilayered pathophysiology that leads to the cardiogenic shock, anchoring on only one portion of BRASH syndrome can lead to a delay in the wholistic treatment needed for these patients and poor outcomes. This emphasizes the importance of having a high index of suspicion for BRASH syndrome when presented with a patient that is exhibiting bradycardia and/or signs of shock when sepsis and other common causes have been determined to be less likely.[Fig f2-cpcem-9-157]

Curiously, our patient had exhibited no renal dysfunction prior to this presentation and admission. Generally, patients suffering from BRASH syndrome will have some aspect of chronic kidney disease^1^ that contributes to the spiraling effects of the syndrome. This case illustrates that even patients without prior kidney disease are at risk for this syndrome. The elderly population in particular are at elevated risk, as these patients are often prescribed atrioventricular nodal blockade medication and are particularly susceptible to prerenal acute kidney injury.

It is important to note that hemodynamic instability can arise from seemingly mild lab abnormalities due to the synergistic nature of this syndrome. While the initial potassium for this patient was technically within normal limits, in the setting of her decreased renal function and atrioventricular nodal blockade this still led to life threatening bradycardia and cardiogenic shock. Subsequent testing showed an elevated potassium, leading to confirmation of the diagnosis, prompt treatment, and a positive outcome for the patient. However, it is easy to dismiss this syndrome if the patient has not yet crossed the threshold into true hyperkalemia, contributing to the difficulty of diagnosis.

While atrioventricular nodal blockade plays an obvious role in decreasing cardiac output via bradycardia, it is unclear if the declining renal function leads to decreased clearance of the atrioventricular blocking agent. For example, metoprolol is commonly found on the medication lists of patients with BRASH syndrome, however declining renal function does not seem to effect the clearance of metoprolol.^2^,^3^ Atenolol, the clearance of which is effected by renal dysfunction,^4^ has also been the atrioventricular blockade agent in prior documented cases of BRASH. Further research is required to ascertain whether the renal dysfunction observed in these patients contributes to more significant bradycardia and/or hypotension, contingent on the specific atrioventricular blocking agent being used.

Determining which aspect of this syndrome actually initiates the cascade of events in a given case can be challenging. The patient in our report had been on beta-blocking medications for years without complication. Renal insult is often the first step towards this syndrome, caused by either renally dosed medications or dehydration in patients with prior kidney disease.^3^,^5^ The patient’s only complaint was fatigue, which is often the only reported symptom in cases of BRASH syndrome. Given the patient’s history of recent surgery and decreased activity level at home, it is reasonable to presume that simple dehydration with resultant pre-renal azotemia is what began her decline, which has been seen in some cases of BRASH syndrome.^5^,^6^

Treatment for BRASH syndrome is focused on the treatment of hyperkalemia and improving cardiac output/renal perfusion via vasopressor support. Treatment of hyperkalemia should follow standard protocols while vasopressors and inotropes can be used concomitantly to transiently restore hemodynamics. Intravenous fluids should also be considered for those patients appearing volume responsive. There is often no need for transcutaneous or transvenous pacing if the syndrome is diagnosed quickly and treated correctly. Atropine is often attempted however given atropine’s mechanism of action (antagonism of muscarinic receptors) is not targeting the atrioventricular nodal blockade nor helping to clear potassium it will have no effect in this syndrome. Overall, treatment of BRASH syndrome is relatively simple; diagnosing this rare and deadly disease state is where the difficulty lies.

## CONCLUSION

A newly emerging clinical entity, BRASH syndrome can be difficult to diagnose and deadly if not treated promptly. While this syndrome is most commonly seen in patients with history of renal disease, this case illustrates that even patients with normal renal function at baseline are at risk. A high index of suspicion must exist, even in patients with normal baseline renal function, in order for this syndrome to be properly diagnosed and treated correctly.

## Figures and Tables

**Figure:**
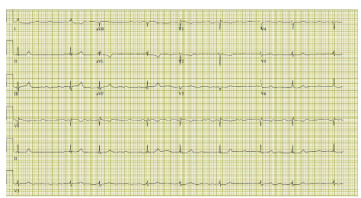
Flowchart illustrating synergistic effects of this syndrome that lead to hemodynamic collapse.

**Image f2-cpcem-9-157:**
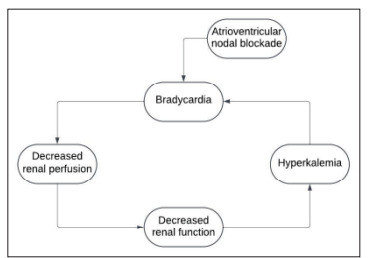
Electrocardiogram taken in Emergency Department depicting atrial fibrillation with slowed ventricular response.
